# Low-Dose Corticosteroid Treatment in Children With *Mycoplasma pneumoniae* Pneumonia: A Retrospective Cohort Study

**DOI:** 10.3389/fped.2020.566371

**Published:** 2020-11-23

**Authors:** Liya Zhang, Lijun Wang, Shanshan Xu, Huajun Li, Caiting Chu, Quanhua Liu, Jia Zhou, Wen Zhang, Lisu Huang

**Affiliations:** ^1^Pediatric Infectious Department, Xinhua Hospital Affiliated to Shanghai Jiao Tong University School of Medicine, Shanghai, China; ^2^Radiological Department, Xinhua Hospital Affiliated to Shanghai Jiao Tong University School of Medicine, Shanghai, China; ^3^Pediatric Respiratory Department, Xinhua Hospital Affiliated to Shanghai Jiao Tong University School of Medicine, Shanghai, China; ^4^Department of Pharmacy, Xinhua Hospital Affiliated to Shanghai Jiao Tong University School of Medicine, Shanghai, China; ^5^Center for Preventive Medical Sciences, Chiba University, Chiba, Japan

**Keywords:** *Mycoplasma pneumoniae* pneumonia (MPP), low-dose corticosteroid, children, refractory, severe

## Abstract

**Background:** The clinical value of corticosteroid treatment in *Mycoplasma pneumoniae* pneumonia (MPP) has been controversial. Our study aimed to identify the effects of low-dose corticosteroids on the recovery of children with MPP.

**Methods:** In this retrospective cohort study, pediatric inpatients with MPP were included from the Shanghai Children's Mycoplasma pneumoniae pneumonia cohort study between August 2014 and July 2019. The multivariable logistic regression and propensity-score matching were used to investigate the effects of low-dose corticosteroid treatment on fever duration after admission, total fever duration, length of hospital stay, C-reactive protein recovery time, and imaging recovery time with the stratification of severe pneumonia, refractory pneumonia, inflammatory biomarkers, pulmonary images, and timing of corticosteroids.

**Results:** There were 548 patients in the corticosteroid group and 337 in the no-corticosteroid group. The corticosteroid group showed severe clinical parameters such as more severe and refractory cases, higher laboratory values, and more abnormal imaging manifestations. The corticosteroid group also showed longer fever duration after admission [odds ratio (OR) = 1.9 (95% CI, 1.2–3.1), *P* = 0.008], longer total fever duration [OR = 1.6 (95% CI, 1.1–2.3), *P* = 0.011], longer hospital stay [OR = 2.8 (95% CI, 1.9–4.0), *P* < 0.001], and longer C-reactive protein (CRP) recovery time [OR = 2.1 (95% CI, 1.1–3.9), *P* = 0.021] in the regression model after the adjustment for severity. Although low-dose corticosteroids were associated with shortened imaging recovery time in patients with high level laboratory values, pulmonary imaging could be completely recovered in both groups. The trend of these results was consistent even after stratifications and a propensity scores matching analysis.

**Conclusions:** Low-dose corticosteroids may not be beneficial in children inpatients with MPP, and further studies on proper treatment modality are needed in the MRMP era.

## Introduction

*Mycoplasma pneumoniae* (MP) is a common cause of community-acquired pneumonia (CAP) in children, accounting for 10–40% (1). A cyclical epidemic of MP infection occurs every 3–7 years (2). *Mycoplasma pneumoniae* pneumonia (MPP) is usually benign and self-limiting, with various clinical manifestations, but some may present a progressive course, which may be life threatening, despite early and adequate treatment. Although macrolides are the first-line antibiotics recommended by all guidelines, the efficacy of antibiotics on MP infection remains controversial with quite few randomized controlled studies (RCTs) available (3). Facing with the increasing rate of macrolide-resistant *Mycoplasma pneumoniae* (MRMP) strains detection and the limitations of alternative antibiotics use on MP infection in children, the need for early immune-modulator treatment (mainly corticosteroids) based on pathogenesis of MPP is of great concern. The severity of MPP seems to depend on the pathogenesis of MP and the host hyperimmune reaction against insults from MP infection (4). The host hyperimmune reaction before the peak of inflammation is believed to be responsible for lung cell injury. The substances produced from injured lung cells induce further inflammation if released into the systemic circulation or near local lesions (5). Therefore, early control of lung injuries from initial hyperactive immune reactions is crucial for the reduction in morbidity and prevention of progression in patients with severe MPP. Since corticosteroids can suppress inflammation via several molecular mechanisms (6), it has been hypothesized that corticosteroid treatment is effective in MPP. However, the effects of corticosteroid treatment on pediatric MPP are also controversial like antibiotics. Some studies have shown that high-dose corticosteroid treatment was effective in children with severe or refractory MPP (7, 8), and others found that early use of corticosteroids was effective (3, 9), while others concluded that corticosteroids were not beneficial in MPP, even refractory cases (10, 11). Even the effectiveness of adjunctive corticosteroid treatment in CAP is unclear until now (12–15). A recent meta-analysis indicated that corticosteroid therapy of CAP may be associated with inhibition of excessive inflammatory response, and modulating cytokines release offers advantages over conventional therapy for relieving clinical symptoms, reducing mortality, and improving prognosis (16). However, most previous studies were retrospective cohort studies with substantial heterogeneity with an inability to determine the etiology of cases of pneumonia, as well as phenotype and pathology (17–19). Thus, we conducted a cohort study with a large sample size to evaluate the effects of adjunctive low-dose corticosteroid treatment for children with MPP, stratified by severe pneumonia, refractory pneumonia, inflammatory biomarkers, pulmonary images, and the timing of corticosteroid treatment.

## Methods

### Patients and Study Design

**Figure 1:**
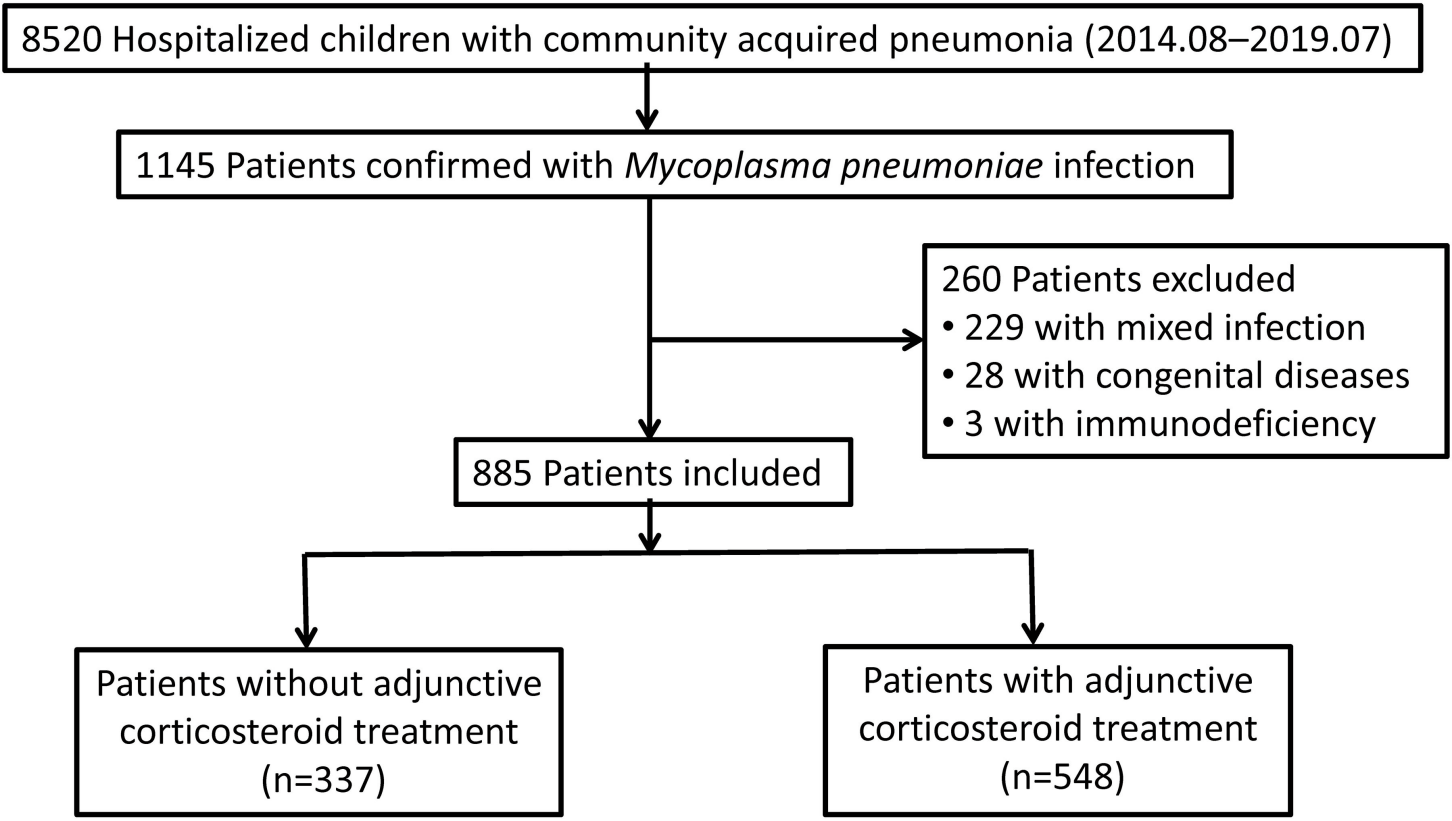
Study flow chart.

The Shanghai Children's *M. pneumoniae* pneumonia cohort was conducted at Xinhua Hospital affiliated to the Shanghai Jiao Tong University School of Medicine from August 2014 to July 2019. Overall, 8,520 children aged between 2 months and 13 years with CAP were hospitalized, and of these, 1,145 were confirmed with MP infection by both positive serologic-test results [MP immunoglobulin (IgM) positive and antibody titer ≥1:160] and positive results on MP polymerase chain reaction testing of pharyngeal swabs (20). Patients with mixed infections of other pathogens, congenital diseases, and immunodeficiency diseases were excluded. Finally, 885 patients were included. All patients received antibiotic treatment of macrolides (21). Patients treated with macrolides only were classified into the no-corticosteroid group (*n* = 337; Figure 1). Patients treated with macrolides and low-dose corticosteroids (intravenous methylprednisolone, 1–2 mg/kg/day for 3–5 days, then replaced by 1 mg/kg/day of oral prednisolone after temperature recovery and tapered and stopped within a week) were classified into the corticosteroid group (*n* = 548) (22). Patients were followed up for disease recovery after discharge by a physician from the Infectious Disease Department or Respiratory Department.

### Data Collection and Definition

Demographic, clinical, laboratory, imaging, and outcome characteristics were abstracted from medical histories. Demographic and clinical characteristics included age, sex, weight, parents' occupations, fever duration before admission, hypoxemia, neurological symptoms, encephalitis, rash, severe pneumonia, treatment, etc. Laboratory data obtained during hospitalization included organ functional index and inflammatory biomarkers [white blood cell count, platelet count, C-reactive protein (CRP), lactic dehydrogenase (LDH), total bilirubin, creatinine, creatine kinase isoenzyme, interleukin (IL)-10]. Imaging characteristics included atelectasis, pleural effusion, consolidation, and multilobar infiltrates based on chest radiograph or low-dose computed tomography. Outcome characteristics included fever duration after admission, total fever duration, length of hospital stay, CRP recovery time, and imaging recovery time.

A group of experts from different disciplines assessed all data collected with regard to the following aspects: respiratory function, extrapulmonary manifestations, illness severity, detection indicators, imaging performances, and disease outcomes. Among them, illness severity referred to the Criteria for CAP Severity of Illness in Children with Community-Acquired Pneumonia (23), and organ failure referred to the Sequential Organ Failure Assessment scoring system. Refractory pneumonia was defined as a case with prolonged fever and aggravation of radiological manifestations despite appropriate antibiotic therapy for 7 days or more (24). CRP recovery time was defined as the number of days that abnormal CRP recovers to normal. Imaging recovery time was defined as the number of days that abnormal imaging performance returns to normal. Multilobar was defined as two lobes and above. There were 358 patients with two comparable imaging data within 1 month. Biomarker stratification index including CRP, LDH, and IL-10, were determined based on previous studies and clinical experience (20, 25, 26). Imaging stratification included atelectasis, pleural effusion, and multilobar consolidations. Missing data included atelectasis (*n* = 50), pleural effusion (*n* = 23), multilobar consolidations (*n* = 6), IL-10 (*n* = 262), CRP (*n* = 11), and LDH (*n* = 124).

The study was approved by the Xinhua Hospital Ethics Committee (XHEC-C-2013-107). Informed consent was obtained by caregivers of all children for their clinical records to be used in this study.

### Statistical Analysis

Categorical variables were presented as counts (percentage). Continuous quantitative variables were presented as mean and standard deviation or median and interquartile range of 25–75%. Comparisons between groups were performed by Kruskal–Wallis-test or Fisher's exact-tests. The effects of corticosteroids were evaluated by different outcome (fever duration after admission, total fever duration, length of hospital stay, CRP recovery time, and imaging recovery time) using Kaplan–Meier analysis. Logistic regression analysis were performed with stratifications by severe pneumonia, refractory pneumonia, inflammatory biomarkers, pulmonary images, and different timing of corticosteroid treatment to evaluate the effects of low-dose corticosteroids on different endpoints, and the 75th percentile were chosen as cutoff points. Results were reported as odds ratio (OR) with 95% confidence interval (CI). Since severity of pneumonia was associated with corticosteroid use, we also performed one to two propensity scores matching to minimize the selection bias. All statistical analyses were performed using Empower R (www.empowerstats.com, X&Y Solutions, Inc., Boston MA, USA) and R software (http://www.T-project.org). A two-tailed *P* < 0.05 was considered to be statistically significant in all analyses.

## Results

Overall, 548 patients received an adjunctive low-dose corticosteroid treatment, while 337 did not. There were 412 boys and 473 girls, with a mean age of 5.2 years. The prevalence of severe pneumonia and refractory pneumonia was 16.3 and 28.7% in all patients, and none died. Low-dose corticosteroids were significantly associated with higher prevalence of hypoxemia, severe pneumonia, refractory pneumonia, multilobar consolidations, atelectasis, and pleural effusion (Table 1). The levels of inflammatory biomarkers, such as IL-10, CRP, and LDH were also significantly higher in the corticosteroid group. The median duration of fever after admission, total fever duration, length of hospital stay, and CRP recovery time in the corticosteroid group were 2, 8, 7, and 5 days, respectively, which were significantly longer than those in the no-corticosteroid group (1, 7, 5, and 4 days, respectively). In contrast, the median imaging recovery time in the corticosteroid group was shorter than that in the no-corticosteroid group (13 vs. 16 days, respectively). Corticosteroid-related adverse effects were observed, and no adverse effects occurred in either group.

**Table 1:** Characteristics of children with *Mycoplasma pneumoniae* pneumonia stratified by adjunctive corticosteroid treatment.

**Variables**	**Total**	**No-corticosteroid**	**Corticosteroid**	***P*-value**
	**(*n* = 885)**	**(*n* = 337)**	**(*n* = 548)**	
Male, *n* (%)	412 (46.6)	144 (42.7)	268 (48.9)	0.074
Age, mean (SD), years	5.2 (2.7)	5.0 (2.7)	5.3 (2.7)	0.188
Weight, mean (SD), kg	21.0 (8.7)	20.6 (8.7)	21.3 (8.7)	0.186
Fever duration before admission, median (IQR), days	6.0 (5.0–8.0)	6.0 (4.0–8.0)	6.0 (5.0–8.0)	0.348
Hypoxemia, *n* (%)	17 (1.9)	2 (0.6)	15 (2.7)	0.024
Neurological symptoms, *n* (%)	10 (1.1)	1 (0.3)	9 (1.6)	0.066
Encephalitis, *n* (%)	2 (0.2)	0 (0.0)	2 (0.4)	0.267
Rash, *n* (%)	2 (0.2)	0 (0.0)	2 (0.4)	0.267
Multilobar infiltrates, *n* (%)	655 (75.6)	228 (70.4)	427 (78.8)	0.005
Multi lobar consolidations, *n* (%)	213 (24.2)	65 (19.4)	148 (27.2)	0.009
Atelectasis, *n* (%)	80 (9.6)	20 (6.6)	60 (11.3)	0.029
Pleural effusion, *n* (%)	145 (16.8)	28 (8.7)	117 (21.7)	<0.001
SOFA ≥ 1, *n* (%)	37 (4.2)	10 (3.0)	27 (4.9)	0.157
Severe pneumonia, *n* (%)	144 (16.3)	28 (8.3)	116 (21.2)	<0.001
Refractory pneumonia, *n*(%)	254 (28.7)	79 (23.4)	175 (31.9)	0.007
White blood cell, median (IQR), 10^9^/L	6.9 (5.3–9.1)	6.9 (5.4–9.3)	6.9 (5.3–8.9)	0.110
Platelet, mean (SD), 10^9^/L	301.9 (107.8)	317.9 (109.6)	292.3 (105.6)	<0.001
CRP, median (IQR), mg/L	11.0 (4.0–25.0)	9.5 (4.0–19.2)	12.0 (4.0–29.0)	0.008
Lactic dehydrogenase, median (IQR), U/L	342.0 (288.0–407.0)	302.0 (258.5–361.0)	365.0 (311.0–445.0)	<0.001
Total bilirubin, mean (SD), μmol/L	5.4 (2.9)	5.5 (3.1)	5.4 (2.7)	0.677
Creatine, mean (SD), μmol/L	31.4 (9.1)	31.5 (7.7)	31.3 (9.9)	0.754
Creatine Kinase Isoenzyme, mean (SD), U/L	21.9 (14.3)	20.9 (11.5)	22.5 (15.8)	0.133
Interleukin-10, median (IQR), pg/mL	2.5 (2.5–9.6)	2.5 (2.5–6.8)	5.2 (2.5–10.8)	0.001
**Prognosis**				
Fever duration after admission, median (IQR), days	1.0 (0.0–3.0)	1.0 (0.0–2.0)	2.0 (1.0–3.0)	<0.001
Total fever duration, median (IQR), days	8.0 (6.0–10.0)	7.0 (5.0–9.0)	8.0 (7.0–11.0)	<0.001
Length of hospital stay, median (IQR), days	7.0 (5.0–8.0)	5.0 (4.0–7.0)	7.0 (6.0–9.0)	0.001
CRP recovery time, median (IQR), days	4.0 (3.0–6.0)	4.0 (3.0–5.0)	5.0 (3.0–6.0)	0.001
Imaging recovery time, median (IQR), days	15.0 (8.0–20.0)	16.0 (10.0–21.0)	13.0 (8.0–19.0)	0.024

*Data are presented as mean (SD) or median (IQR) or n (%)*.

*SD, standard deviation; IQR, interquartile range; SOFA, sequential organ failure assessment score; CRP, C-reactive protein*.

**Figure 2:**
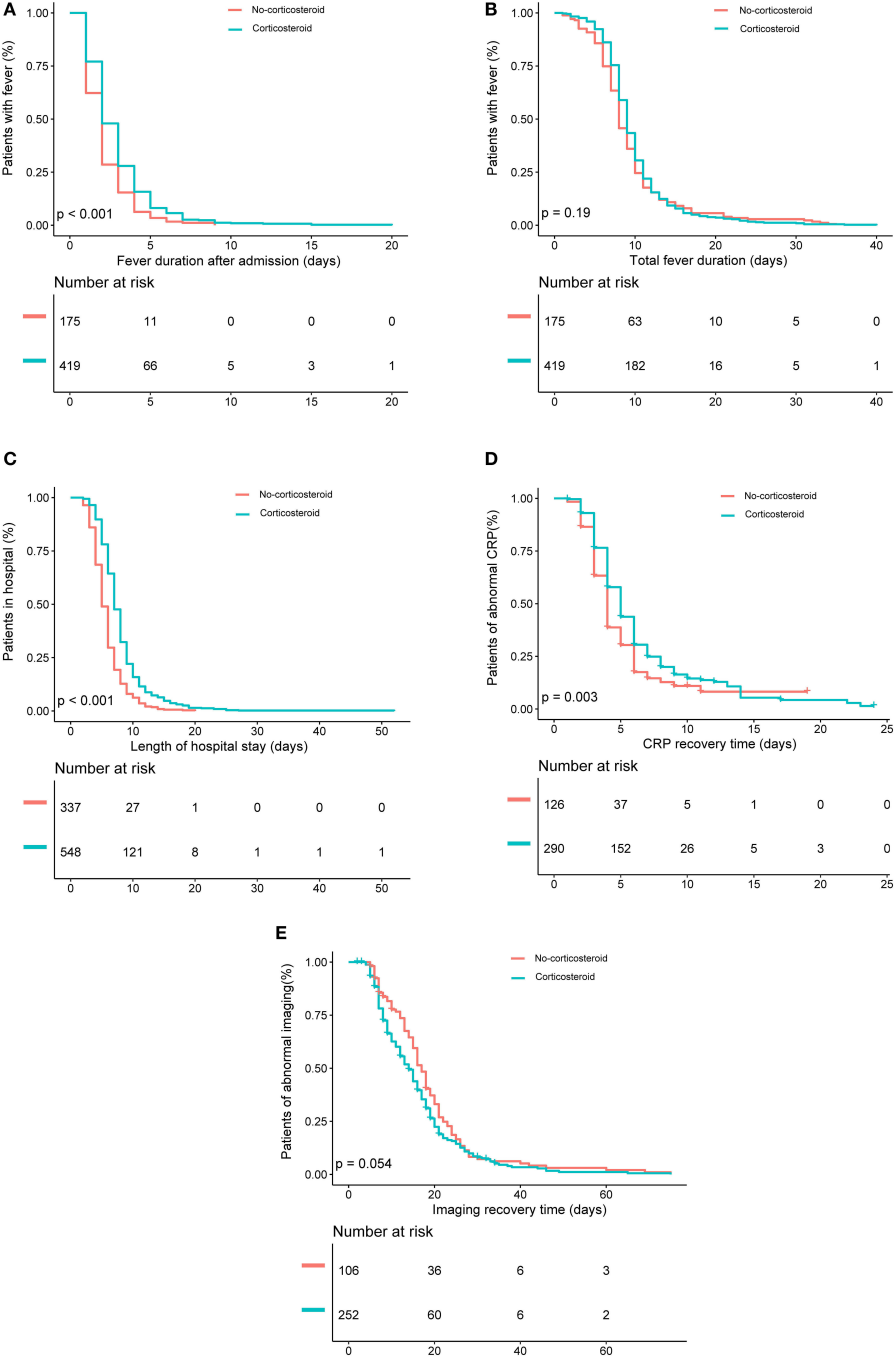
Kaplan–Meier analysis of the effects of low-dose corticosteroid treatment on **(A)** fever duration after admission, **(B)** total fever duration, **(C)** length of hospital stay, **(D)** C-reactive protein (CRP) recovery time, and **(E)** imaging recovery time in the entire patient sample.

Kaplan–Meier analysis showed that the corticosteroid group were significantly associated with longer fever duration after admission (Figure 2A), longer hospital stay (Figure 2C), and increased CRP recovery time (Figure 2D). However, no significant between-group differences occurred in total fever duration and imaging recovery time on Kaplan–Meier analysis (*P* = 0.19 and *P* = 0.054; Figures 2B,E). Logistic regression analyses showed that the corticosteroid group was associated with increased odds to experience a fever for longer than 3 days after admission (OR, 1.9; 95% CI, 1.2–3.1), a total fever longer than 10 days (OR, 1.6; 95% CI, 1.1–2.3), a hospital stay longer than 8 days (OR, 2.8; 95% CI, 1.9–4.0), and a CRP recovery time longer than 6 days (OR, 2.1; 95% CI, 1.1–3.9) (Table 2) after adjustments. However, there was no significant association between low-dose corticosteroid treatment and imaging recovery time after adjustments.

**Table 2:** Logistic regression analysis for the effects of adjunctive corticosteroid treatment in *Mycoplasma pneumoniae* pneumonia patients.

**Variables**	**Total**	**No-corticosteroid**	**Corticosteroid**	**OR****^a^**(**95%CI)**	***P*-value**
**Overall**	***n*** **=** **885**	***n*** **=** **337**	***n*** **=** **548**		
Fever duration after admission > 75th (3 days)	144 (24.2)	27 (15.4)	117 (27.9)	1.9 (1.2, 3.1)	0.008
Total fever duration > 75th (10 days)	198 (22.4)	55 (16.3)	143 (26.1)	1.6 (1.1, 2.3)	0.011
Length of hospital stay > 75th (8 days)	220 (24.9)	43 (12.8)	177 (32.3)	2.8 (1.9, 4.0)	<0.001
CRP recovery time > 75th (6 days)	91 (24.9)	15 (14.6)	76 (29.0)	2.1 (1.1, 3.9)	0.021
Imaging recovery time > 75th (20 days)	104 (29.1)	39 (36.8)	65 (25.8)	0.6 (0.4, 1.0)	0.063
**Patients with severe pneumonia**	***n*** **=** **144**	***n*** **=** **28**	***n*** **=** **116**		
Fever duration after admission > 75th (4 days)	30 (25.6)	3 (15.8)	27 (27.6)	2.2 (0.6, 8.2)	0.253
Total fever duration > 75th (12 days)	30 (20.8)	5 (17.9)	25 (21.6)	1.1 (0.4, 3.3)	0.835
Length of hospital stay > 75th (12 days)	29 (20.1)	3 (10.7)	26 (22.4)	2.2 (0.6, 7.8)	0.242
CRP recovery time > 75th (8 days)	24 (27.3)	2 (15.4)	22 (29.3)	2.1 (0.4, 10.6)	0.357
Imaging recovery time > 75th (19 days)	19 (25.3)	2 (22.2)	17 (25.8)	1.2 (0.2, 6.2)	0.853
**Patients with refractory pneumonia**	***n*** **=** **254**	***n*** **=** **79**	***n*** **=** **175**		
Fever duration after admission > 75th (4 days)	57 (27.8)	8 (14.8)	49 (32.5)	2.5 (1.1, 5.8)	0.032
Total fever duration > 75th (13 days)	63 (24.8)	16 (20.3)	47 (26.9)	1.4 (0.7, 2.7)	0.331
Length of hospital stay > 75th (11 days)	50 (19.7)	8 (10.1)	42 (24.0)	2.0 (0.9, 4.8)	0.111
CRP recovery time > 75th (7 days)	32 (22.7)	4 (11.8)	28 (26.2)	2.0 (0.6, 6.7)	0.252
Imaging recovery time > 75th (21 days)	23 (23.7)	6 (24.0)	17 (23.6)	1.2 (0.4, 3.9)	0.741
**Patients with CRP** **>** **75th (25 mg/L)**	***n*** **=** **217**	***n*** **=** **64**	***n*** **=** **153**		
Fever duration after admission > 75th (4 days)	29 (15.5)	4 (8.5)	25 (17.9)	1.9 (0.6, 5.9)	0.291
Total fever duration > 75th (11 days)	41 (18.9)	5 (7.8)	36 (23.5)	2.9 (1.1, 8.1)	0.039
Length of hospital stay > 75th (10 days)	44 (20.3)	10 (15.6)	34 (22.2)	1.1 (0.4, 2.5)	0.906
CRP recovery time > 75th (6 days)	56 (33.3)	8 (18.6)	48 (38.4)	2.4 (1.0, 5.6)	0.054
Imaging recovery time > 75th (20 days)	23 (24.7)	6 (27.3)	17 (23.9)	0.7 (0.2, 2.2)	0.513
**Patients with LDH** **>** **75th (407 U/L)**	***n*** **=** **190**	***n*** **=** **30**	***n*** **=** **160**		
Fever duration after admission > 75th (4 days)	33 (20.5)	0 (0.0)	33 (23.4)	Inf. (0.0, Inf.)	0.990
Total fever duration > 75th (12 days)	47 (24.7)	10 (33.3)	37 (23.1)	0.5 (0.2, 1.2)	0.116
Length of hospital stay > 75th (10 days)	43 (22.6)	4 (13.3)	39 (24.4)	1.2 (0.4, 4.0)	0.728
CRP recovery time > 75th (7 days)	27 (23.7)	1 (9.1)	26 (25.2)	2.2 (0.2, 19.2)	0.494
Imaging recovery time > 75th (19 days)	20 (27.0)	5 (55.6)	15 (23.1)	0.2 (0.0, 0.8)	0.022
**Patients with IL-10** **>** **75th (9.6 pg/ml)**	***n*** **=** **156**	***n*** **=** **42**	***n*** **=** **114**		
Fever duration after admission > 75th (4 days)	22 (17.2)	2 (6.7)	20 (20.4)	3.3 (0.7, 15.8)	0.136
Total fever duration > 75th (12 days)	34 (21.8)	7 (16.7)	27 (23.7)	1.4 (0.5, 3.7)	0.497
Length of hospital stay > 75th (9 days)	34 (21.8)	3 (7.1)	31 (27.2)	2.3 (0.6, 8.7)	0.227
CRP recovery time > 75th (7 days)	18 (26.1)	2 (13.3)	16 (29.6)	2.1 (0.4, 12.0)	0.407
Imaging recovery time > 75th (21 days)	14 (24.1)	6 (40.0)	8 (18.6)	0.2 (0.0, 1.2)	0.074
**Patients with pleural effusion**	***n*** **=** **145**	***n*** **=** **28**	***n*** **=** **117**		
Fever duration after admission > 75th (4 days)	26 (21.8)	2 (10.0)	24 (24.2)	3.3 (0.7, 15.7)	0.135
Total fever duration > 75th (12 days)	29 (20.0)	5 (17.9)	24 (20.5)	1.1 (0.4, 3.3)	0.861
Length of hospital stay > 75th (12 days)	28 (19.3)	4 (14.3)	24 (20.5)	1.4 (0.4, 4.6)	0.561
CRP recovery time > 75th (8 days)	23 (27.4)	2 (15.4)	21 (29.6)	2.0 (0.4, 10.1)	0.414
Imaging recovery time > 75th (19 days)	18 (24.0)	1 (12.5)	17 (25.4)	2.5 (0.3, 22.6)	0.407
**Patients with multilobar consolidations**	***n*** **=** **213**	***n*** **=** **65**	***n*** **=** **148**		
Fever duration after admission > 75th (3 days)	52 (33.3)	6 (17.1)	46 (38.0)	2.8 (1.1, 7.6)	0.037
Total fever duration > 75th (11 days)	51 (23.9)	9 (13.8)	42 (28.4)	2.1 (0.9, 4.7)	0.081
Length of hospital stay > 75th (9 days)	49 (23.0)	7 (10.8)	42 (28.4)	2.8 (1.1, 6.9)	0.029
CRP recovery time > 75th (7 days)	28 (23.7)	4 (16.0)	24 (25.8)	1.3 (0.4, 4.6)	0.645
Imaging recovery time > 75th (21 days)	22 (24.2)	9 (32.1)	13 (20.6)	0.5 (0.2, 1.5)	0.251

*Data are presented as no. (%), OR, and 95% CI*.

*OR, odds ratio; CI, confidence interval; CRP, C-reactive protein; LDH, lactic dehydrogenase; IL-10, interleukin-10*.

a *Adjusted for age, sex, and severe pneumonia*.

To better investigate the effects of adjunctive low-dose corticosteroid treatment on recovery of children with MPP, analyses in severe pneumonia cases, refractory pneumonia cases, cases with high level inflammatory biomarkers, and cases with abnormal imaging findings (Table 2) were performed. The trend of the results was the same as before stratification, except a significant decreased odds to experience an imaging recovery time longer than 19 days in patients with high level of LDH treated by corticosteroids (OR, 0.2; 95% CI, 0.0–0.8).

The effects of timing of low-dose corticosteroid treatment were also investigated by logistic regression analyses with stratification by days after the fever onset (1–5 days, 6–10 days, >10 days) (Table 3). No statistically significant benificial effect on each endpoint in any of the subgroups of corticosteroids was observed except a significantly decreasing imaging recovery time in the subgroup of corticosteroid treatment debut 6–10 days after the fever onset (OR, 0.6; 95% CI, 0.3–1.0).

**Table 3:** Logistic regression analysis for the effects of adjunctive corticosteroid treatment stratified by medication time in *Mycoplasma pneumoniae* pneumonia patients.

**Variables**	**No-corticosteroid**	**Corticosteroid (1–5 days)**			**Corticosteroid (6–10 days)**			**Corticosteroid (>10 days)**		
	**(*n* = 337)**	**(*****n*** **=** **117)**			**(*****n*** **=** **319)**			**(*****n*** **=** **83)**		
	***n* (%)**	***n* (%)**	**OR**^**a**^**(95%CI)**	***P*-value**	***n* (%)**	**OR (95%CI)**	***P*-value**	***n* (%)**	**OR (95%CI)**	***P*-value**
Fever duration after admission > 75th (3 days)	27 (15.4)	19 (23.8)	1.5 (0.8, 3.0)	0.220	73 (27.0)	1.9 (1.2, 3.2)	0.011	22 (35.5)	2.5 (1.2, 4.9)	0.010
Total fever duration > 75th (10 days)	55 (16.3)	14 (12.0)	0.6 (0.3, 1.2)	0.129	63 (19.7)	1.1 (0.7, 1.7)	0.661	62 (74.7)	13.7 (7.7, 24.5)	<0.001
Length of hospital stay > 75th (8 days)	43 (12.8)	26 (22.2)	1.7 (1.0, 3.0)	0.063	115 (36.1)	3.4 (2.2, 5.1)	<0.001	32 (38.6)	3.5 (2.0, 6.2)	<0.001
CRP recovery time > 75th (6 days)	15 (14.6)	10 (21.7)	1.5 (0.6, 3.6)	0.420	48 (30.2)	2.2 (1.2, 4.3)	0.016	15 (32.6)	2.4 (1.0, 5.6)	0.040
Imaging recovery time > 75th (20 days)	39 (36.8)	14 (32.6)	0.9 (0.4, 1.8)	0.683	37 (23.6)	0.6 (0.3, 1.0)	0.036	11 (27.5)	0.7 (0.3, 1.5)	0.337

*Data are presented as no. (%), OR, and 95% CI*.

*OR, odds ratio; CI, Confidence interval; CRP, C-reactive protein*.

a *Adjusted for age, sex, and severe pneumonia*.

To minimize the selection bias, we matched severity of pneumonia, LDH, and CRP using propensity scores. After matching, there were no significant differences between the baseline of patients with and without low-dose corticosteroid treatment (Appendix Table 1). The median duration of fever after admission and length of hospital stay in the corticosteroid group were 2 and 8 days, respectively, which were significantly longer than those in the no-corticosteroid group (1 and 6 days, respectively). The trend of results was consistent with the results before PS matching in logistic regression analyses with stratification by clinical parameters (Appendix Table 2) and medication time (Appendix Table 3).

## Discussion

Although often prescribed clinically, the effects of low-dose corticosteroid treatment in disease recovery in children with MPP remains inconsistent. Different from previous studies, we found that low-dose corticosteroid treatment was associated with significantly increased fever duration, hospital stay, and CRP recovery time. Despite possible shortening of the imaging recovery time, all patients recovered completely albeit recovery required moderately longer in the no-corticosteroid group. Therefore, low-dose corticosteroid treatment may not benefit children with MPP. Our conclusion is consistent with Japanese studies in MPP. A retrospective study found that adjunctive corticosteroid treatment was associated with increases in length of stay, hyperglycemia requiring insulin treatment, and drug costs in adults with MPP (27). A study of children inpatients with MPP found that corticosteroids associated with longer hospital stay, higher total hospitalization costs, and higher risk of 30-day readmission (10). Neither study proved the benefits of corticosteroids in MPP and reached the same conclusion as our study that low-dose corticosteroids may increase the length of hospital stay. Longer hospital stay often leads to more drug administrations, which also increases the cost of hospitalization; therefore, we did not evaluate hospitalization costs. There were no adverse effects in both groups, which might be related with the low-dose administration of corticosteroids.

In fact, limited data with good evidence are available for corticosteroid use in the treatment of pediatric MPP. A recent meta-analysis found that the early treatment of corticosteroids may be beneficial; however, in these studies with a generally small sample size of <100 patients each, significant statistical heterogeneity in many outcomes including length of stay, pulmonary rales improvement time, cough disappearance time, temperature recovery time, and pulmonary shadow absorption time, with the *I*^2^ higher than 90% (8). Besides, these studies only included cases with severe pneumonia, and the indications, timing of corticosteroid administration, and age of patients all varied greatly. With a larger sample size, we applied the same strategy to every patient with MPP, and further, we performed stratification by clinical parameters and propensity score matching to minimize confounders. In the clinical setting, most pediatricians accept that corticosteroids may be effective for severe or refractory MPP, which has also been mentioned in the guidelines. However, no benefits of low-dose corticosteroid treatment were found in the severe pneumonia subgroup or refractory pneumonia subgroup after stratifications. Therefore, we suggest clinicians to be more prudent for low-dose corticosteroid treatment in severe or refractory MPP. Whether high-dose corticosteroid treatment could benefit children inpatients with MPP needed further evaluations.

The clinical value of adjuvant corticosteroid treatment in CAP has also been controversial for a long time. Previous RCTs aimed to assess the effects of corticosteroids in patients with CAP who were not in intensive care units (ICUs) and drew different conclusions. Some concluded that corticosteroids did not improve outcomes (28), while others concluded that corticosteroids reduced length of hospital stay (29, 30). Effects of corticosteroids in severe CAP are of greater concern. Methylprednisolone has been reported to reduce fever duration in children with severe CAP (31). A recent meta-analysis including 10 RCTs comprising 665 patients with severe CAP concluded that adjunctive corticosteroids yielded favorable outcomes as evidenced by decreased all-cause mortality, incidence of septic shock, and requirement for mechanical ventilation without increasing risk of adverse events (32). These outcomes for severe CAP focusing on ICU assessment were different from ours. We found that children inpatients with MPP rarely had actual organ damage or needed mechanical ventilation or ICU transport even meeting the criteria of severe pneumonia in our study. Furthermore, there were no fatal cases in either group of our study. Therefore, we found no benefits of low-dose corticosteroid use for these inpatients based on severe outcomes. It is possible that MPP is a self-limiting disease regardless of severity.

In addition to disease severity, researchers have also focused on inflammatory responses. Corticosteroid therapy in patients with severe CAP and high inflammatory responses was associated with less treatment failure, lower inflammatory responses (33), shortened time to clinical stability (defined as improvement in chest X-ray and normalization of temperature, respiratory rate, and inflammatory markers), and especially less radiological progression (34). A high inflammatory response is thought to be the basis for effective corticosteroid therapy in severe CAP patients (35). Two large RCTs investigating septic shock showed that low-dose corticosteroids had a limited role in patients with sepsis and CAP who were not in septic shock (36). The results suggested that the degree and severity of high inflammatory response might affect the treatment effects of corticosteroids. In our study, a majority of patients were not in septic shock. We stratified patients by CRP, LDH, and IL-10 as inflammatory biomarkers to evaluate whether the inflammatory response influences the effects of corticosteroids in children with MPP. We found that low-dose corticosteroids did not improve the outcomes except a significant shortened imaging recovery time in patients with high level of LDH. However, all patients with abnormal imaging recovered completely. The effects of immunomodulators (such as corticosteroids) on immune cells is dose dependent. We speculate that high-dose corticosteroids might help more than early imaging recovery from the disease.

We also analyzed the relationship between imaging performance and benefits of low-dose corticosteroid treatment. Dexamethasone was reported as a safe and effective adjunctive therapy in CAP patients with parapneumonic pleural effusion (37). We stratified patients by different imaging manifestations including pleural effusion and found no benefit of low-dose corticosteroid treatment for MPP recovery. A study using an experimental mouse model of MP respiratory infection concluded that adjuvant corticosteroid use decreased levels of histological signs of lung inflammation (38). It may explain our findings that corticosteroids associated with improvement on imaging recovery. In clinical practice, we discharge patients usually after the acute phase of disease without waiting for the recovery of imaging abnormalities. Therefore, imaging abnormalities generally do not change the treatment process. Thus, the improvement on imaging recovery is less meaningful compared with increased fever duration and hospital stay. Therefore, it may not be necessary to use low-dose corticosteroids to achieve imaging remission in children with MPP.

We also analyzed the relationship between administration time of low-dose corticosteroid treatment and its possible benefit. Previous studies concluded that early corticosteroid therapy favored a better outcome in MPP (3, 9, 39); therefore, the timing of administration should be considered for corticosteroid use. Due to the limitation of retrospective study, we can only group the patients according to the days after fever onset. However, we did not find significant difference between the low-dose corticosteroid groups with different timing of administration. Whether the use of corticosteroids in early stage of MP infection could improve the outcomes needs further evaluation.

Based on our data, low-dose corticosteroid treatment may not be beneficial in some clinical parameters, such as severity of illness, high inflammatory factors, and abnormal imaging manifestations, in the treatment of MPP in children. Why previous RCTs and cohort studies evaluating the use of corticosteroids in CAP drew different conclusions might be related to the pathogenic complexity of CAP. The underlying mechanism that could explain our findings is unclear. We hypothesize that it might be the interaction between MP pathology and corticosteroid immunomodulatory activity. Pathogenesis of MP is complicated and involves several different pathways: cytoadherence to respiratory tract epithelium, intracellular localization, direct cytotoxicity, activation of the inflammatory cascade through toll-like receptors leading to inflammatory cytokine-mediated tissue injury, community-acquired respiratory distress syndrome toxin, and inflammasome activation (40). Corticosteroids may suppress inflammation by inhibiting activated inflammatory gene transcription and post-transcriptional effects (6) and may also present some side effects, such as broad immunosuppression and activation of latent viruses (41). Corticosteroids can improve vascular permeability, reduce immune-active cells around inflammatory lesions, and reduce vasodilation. As a consequence, corticosteroids not only may improve pulmonary imaging but also may accelerate the dissemination of MP, which is usually located in the airway, causing an increase in fever duration. In *Chlamydia pneumoniae* pneumonia, it has been well-documented that corticosteroid use drove *C. pneumoniae* out of a persistent state into active replication and resulted in exacerbation of the inflammatory process (42).

In our study, several limitations should be considered. First, the characteristics of “retrospective” made our study have some inevitable bias. One of the most important bias was patient selection bias. The proportion of severe or refractory pneumonia was significantly higher in the corticosteroid group, since mild pneumonia was usually treated in the community. We generated a propensity score-matching sample to minimize the selection bias and balance the baseline of population. After propensity score matching, there were no significant differences between the baseline of patients with and without low-dose corticosteroid treatment. The results of the analysis are consistent with those before matching. A part of the children were not tested for IL-10, which resulted in missing data. We use propensity score matching to minimize the impact of missing data, instead of data imputation. For the diagnostics of MP infection, we tested serum-specific IgM antibody and MP DNA of pharyngeal swab samples by PCR because of the difficulty to draw blood several times for children patients in clinical practice.

Second, all patients were treated by low-dose corticosteroids. Although MPP is a self-limiting disease, the severity of lung injury in MP infection may be associated with the extent of host immune reaction against the amount of etiological or inflammation-inducing substances in the acute stage, and the effect of immune modulators on immune cells is also dose dependent (43). Thus, we could presume that patients with severe MP pneumonia have more severe immune disturbance and respond to the higher-dose immune modulators because of the same immune pathogenesis of mild and severe MP pneumonia (8). Furthermore, a recent study showed that antibiotics may have a limited effect on MP infection, and early and adjustment of corticosteroid dose according to severity of the disease was more effective for reducing morbidity, preventing disease progression, and reducing adverse reaction of corticosteroids (3). Thus, low-dose corticosteroids might have a limitation to evaluate the effects, and further studies are needed.

Third, we did not determine the macrolide resistance for all of the patients, as it is not a determinant of clinical severity in MRMP pneumonia (44). However, patients infected with MRMP had a longer febrile period, length of hospital stay, antibiotic drug courses, and defervescence time after macrolide treatment compared with patients infected with macrolide-sensitive *Mycoplasma pneumoniae* (MSMP) (45). In the MRMP era, problems to use antibiotics for patients with MPP in children become more obvious, and corticosteroids might be critical for reducing morbidity on MP infection. Therefore, our results might be affected to some extent by MP resistance.

In conclusion, although we found that low-dose corticosteroid treatment may not be beneficial in some clinical parameters in children inpatients with MPP, further studies on proper treatment modality are needed in the MRMP era.

## Data Availability Statement

The raw data supporting the conclusions of this article will be made available by the authors, without undue reservation.

## Ethics Statement

The studies involving human participants were reviewed and approved by Xinhua Hospital Ethics Committee (XHEC-C-2013-107). Written informed consent to participate in this study was provided by the participants' legal guardian/next of kin.

## Author Contributions

LZ conceptualized and designed the study, drafted the initial manuscript, and reviewed and revised the manuscript. LW, SX, and HL collected data, carried out the initial analyses, and reviewed and revised the manuscript. CC, QL, JZ, and WZ coordinated and supervised data collection and critically reviewed the manuscript for important intellectual content. LH designed the study and reviewed and revised the manuscript. All authors approved the final manuscript as submitted and agree to be accountable for the content of the work.

## Conflict of Interest

The authors declare that the research was conducted in the absence of any commercial or financial relationships that could be construed as a potential conflict of interest.

## Abbreviations

MP: *Mycoplasma pneumoniae*
MPP: *Mycoplasma pneumoniae* pneumonia
CAP: community acquired pneumonia
RCT: randomized controlled trial
MRMP: macrolide-resistant *Mycoplasma pneumoniae*
CRP: C-reactive protein
LDH: lactic dehydrogenase
IL: interleukin
OR: odds ratio
CI: confidence interval
ICU: intensive care unit
MSMP: macrolide-sensitive *Mycoplasma pneumoniae*.

## References

[1] Yan Y Wei Y Jiang W Hao C The clinical characteristics of corticosteroid-resistant refractory *Mycoplasma pneumoniae* pneumonia in children. Sci Rep. (2016) 6:39929 10.1038/srep39929 28008989 PMC5180238

[2] Lee KY Pediatric respiratory infections by *Mycoplasma pneumoniae*. Expert Rev Anti Infect Ther. (2008) 6:509–21. 10.1586/14787210.6.4.509 18662117

[3] Yang EA Kang HM Rhim JW Kang JH Lee KY Early corticosteroid therapy for *Mycoplasma pneumoniae* pneumonia irrespective of used antibiotics in children. J Clin Med. (2019) 8:726 10.20944/preprints201904.0070.v1 PMC6572103 31121867

[4] Saraya T Kurai D Nakagaki K Sasaki Y Niwa S Tsukagoshi H. Novel aspects on the pathogenesis of *Mycoplasma pneumoniae* pneumonia and therapeutic implications. Front Microbiol. (2014) 5:410. 10.3389/fmicb.2014.00410 25157244 PMC4127663

[5] Lee KY. Pneumonia, acute respiratory distress syndrome, and early immune-modulator therapy. Int J Mol Sci. (2017) 18:388. 10.3390/ijms18020388 28208675 PMC5343923

[6] Barnes PJ. Glucocorticosteroids: current and future directions. Br J Pharmaco. (2011) 163:29–43. 10.1111/j.1476-5381.2010.01199.x 21198556 PMC3085866

[7] Okumura T Kawada JI Tanaka M Narita K Ishiguro T Hirayama Y. Comparison of high-dose and low-dose corticosteroid therapy for refractory *Mycoplasma pneumoniae* pneumonia in children. J Infect Chemother. (2019) 25:346–50. 10.1016/j.jiac.2019.01.003 30718192

[8] Sun LL Ye C Zhou YL Zuo SR Deng ZZ Wang CJ. Meta-analysis of the clinical efficacy and safety of high- and low-dose methylprednisolone in the treatment of children with severe *Mycoplasma pneumoniae* pneumonia. Pediatr Infect Dis J. (2020) 39:177–83. 10.1097/INF.0000000000002529 31738328

[9] Huang L Gao X Chen M. Early treatment with corticosteroids in patients with *Mycoplasma pneumoniae* pneumonia: a randomized clinical trial. J Trop Pediatr. (2014) 60:338–42. 10.1093/tropej/fmu022 24710342

[10] Okubo Y Michihata N Morisaki N Uda K Miyairi I Ogawa Y. Recent trends in practice patterns and impact of corticosteroid use on pediatric *Mycoplasma pneumoniae*-related respiratory infections. Respir Investig. (2018) 56:158–65. 10.1016/j.resinv.2017.11.005 29548654

[11] Ha SG Oh KJ Ko KP Sun YH Ryoo E Tchah H. Therapeutic efficacy and safety of prolonged macrolide, corticosteroid, doxycycline, and levofloxacin against macrolide-unresponsive *Mycoplasma pneumoniae* pneumonia in children. J Korean Med Sci. (2018) 33:e268. 10.3346/jkms.2018.33.e268 30344461 PMC6193889

[12] Polverino E Cilloniz C Dambrava P Gabarrus A Ferrer M Agusti C. Systemic corticosteroids for community-acquired pneumonia: reasons for use and lack of benefit on outcome. Respirology. (2013) 18:263–71. 10.1111/resp.12013 23134361

[13] Siemieniuk RA Meade MO Alonso-Coello P Briel M Evaniew N Prasad M. Corticosteroid therapy for patients hospitalized with community-acquired pneumonia: a systematic review and meta-analysis. Ann Intern Med. (2015) 163:519–28. 10.7326/M15-0715 26258555

[14] Stern A Skalsky K Avni T Carrara E Leibovici L Paul M. Corticosteroids for pneumonia. Cochrane Database Syst Rev. (2017) 12:CD007720. 10.1002/14651858.CD007720.pub3 29236286 PMC6486210

[15] Briegel J Bein T Mohnle P. Update on low-dose corticosteroids. Curr Opin Anaesthesiol. (2017) 30:186–91. 10.1097/ACO.0000000000000442 28118166

[16] Wan YD Sun TW Liu ZQ Zhang SG Wang LX Kan QC. Efficacy and safety of corticosteroids for community-acquired pneumonia: a systematic review and meta-analysis. Chest. (2016) 149:209–19. 10.1378/chest.15-1733 26501852

[17] Garcia-Vidal C Calbo E Pascual V Ferrer C Quintana S Garau J. Effects of systemic steroids in patients with severe community-acquired pneumonia. Eur Respir J. (2007) 30:951–6. 10.1183/09031936.00027607 17690125

[18] Chon GR Lim CM Koh Y Hong SB. Analysis of systemic corticosteroid usage and survival in patients requiring mechanical ventilation for severe community-acquired pneumonia. J Infect Chemother. (2011) 17:449–55. 10.1007/s10156-010-0196-8 21165755

[19] Tagami T Matsui H Horiguchi H Fushimi K Yasunaga H. Low-dose corticosteroid use and mortality in severe community-acquired pneumonia patients. Eur Respir J. (2015) 45:463–72. 10.1183/09031936.00081514 25323232

[20] Zhang Y Zhou Y Li S Yang D Wu X Chen Z. The clinical characteristics and predictors of refractory *Mycoplasma pneumoniae* pneumonia in children. PLoS ONE. (2016) 11:e0156465. 10.1371/journal.pone.0156465 27227519 PMC4882022

[21] Subspecialty Group of Respiratory Diseases TSoPCMA Editorial Board CJoP Guidelines for management of community acquired pneumonia in children (the revised edition of 2013) (I). Zhonghua er ke za zhi. (2013) 51:745–52. 10.3760/cma.j.issn.0578-1310.2013.10.006 24406226

[22] Lu A Wang L Zhang X Zhang M Combined treatment for child refractory *Mycoplasma pneumoniae* pneumonia with ciprofloxacin and glucocorticoid. Pediatr Pulmonol. (2011) 46:1093–7. 10.1002/ppul.21481 21698787

[23] Bradley JS Byington CL Shah SS Alverson B Carter ER Harrison C The management of community-acquired pneumonia in infants and children older than 3 months of age: clinical practice guidelines by the Pediatric Infectious Diseases Society and the Infectious Diseases Society of America. Clinical Infect Dis. (2011) 53:e25–76. 10.1093/cid/cir531 21880587 PMC7107838

[24] Subspecialty Group of Respiratory Diseases TSoP Chinese Medical Association The Editorial Board CJoP Guidelines for management of community acquired pneumonia in children (the revised edition of 2013) (II). Zhonghua er ke za zhi. (2013) 51:856–62. 10.3760/cma.j.issn.0578-1310.2013.11.012 24484563

[25] Lu AZ Wang CK Zhang XB Wang LB Qian LL. Lactate dehydrogenase as a biomarker for prediction of refractory *Mycoplasma pneumoniae* pneumonia in Children. Resp Care. (2015) 60:1469–75. 10.4187/respcare.03920 26060318

[26] Ding S Wang X Chen W Fang Y Liu B Liu Y. Decreased lnterleukin-10 responses in children with severe *Mycoplasma pneumoniae* pneumonia. PLoS ONE. (2016) 11:e0146397. 10.1371/journal.pone.0146397 26751073 PMC4708986

[27] Tashiro M Fushimi K Kawano K Takazono T Saijo T Yamamoto K. Adjunctive corticosteroid therapy for inpatients with *Mycoplasma pneumoniae* pneumonia. BMC Pulm Med. (2017) 17:219. 10.1186/s12890-017-0566-4 29284447 PMC5747073

[28] Snijders D Daniels JM de Graaff CS van der Werf TS Boersma WG. Efficacy of corticosteroids in community-acquired pneumonia: a randomized double-blinded clinical trial. Am J Respir Crit Care Med. (2010) 181:975–82. 10.1164/rccm.200905-0808OC 20133929

[29] Meijvis SC Hardeman H Remmelts HH Heijligenberg R Rijkers GT van Velzen-Blad H. Dexamethasone and length of hospital stay in patients with community-acquired pneumonia: a randomised, double-blind, placebo-controlled trial. Lancet. (2011) 377:2023–30. 10.1016/S0140-6736(11)60607-7 21636122

[30] Blum CA Nigro N Briel M Schuetz P Ullmer E Suter-Widmer I. Adjunct prednisone therapy for patients with community-acquired pneumonia: a multicentre, double-blind, randomised, placebo-controlled trial. Lancet. (2015) 385:1511–8. 10.1016/S0140-6736(14)62447-8 25608756

[31] Nagy B Gaspar I Papp A Bene Z Nagy B Jr Voko Z Efficacy of methylprednisolone in children with severe community acquired pneumonia. Pediatr Pulmonol. (2013) 48:168–75. 10.1002/ppul.22574 22588852

[32] Jiang S Liu T Hu Y Li R Di X Jin X Efficacy and safety of glucocorticoids in the treatment of severe community-acquired pneumonia: a meta-analysis. Medicine. (2019) 98:e16239 10.1097/MD.0000000000016239 31261585 PMC6616855

[33] Torres A Sibila O Ferrer M Polverino E Menendez R Mensa J Effect of corticosteroids on treatment failure among hospitalized patients with severe community-acquired pneumonia and high inflammatory response: a randomized clinical trial. JAMA. (2015) 313:677–86. 10.1001/jama.2015.88 25688779

[34] Prina E Ceccato A Torres A New aspects in the management of pneumonia. Crit Care. (2016) 20:267 10.1186/s13054-016-1442-y 27716262 PMC5045574

[35] Torres A Ferrer M. What's new in severe community-acquired pneumonia? Corticosteroids as adjunctive treatment to antibiotics. Intensive Care Med. (2016) 42:1276–8. 10.1007/s00134-015-4042-4 26370691 PMC7095221

[36] Marik PE. The role of glucocorticoids as adjunctive treatment for sepsis in the modern era. Lancet Respir Med. (2018) 6:793–800. 10.1016/S2213-2600(18)30265-0 30006071

[37] Tagarro A Otheo E Baquero-Artigao F Navarro ML Velasco R Ruiz M. Dexamethasone for parapneumonic pleural effusion: a randomized, double-blind, clinical trial. J Pediatr. (2017) 185:117–23.e6. 10.1016/j.jpeds.2017.02.043 28363363

[38] Tagliabue C Salvatore CM Techasaensiri C Mejias A Torres JP Katz K. The impact of steroids given with macrolide therapy on experimental *Mycoplasma pneumoniae* respiratory infection. J Infect Dis. (2008) 198:1180–8. 10.1086/591915 18717637 PMC2562003

[39] Yang EA Lee KY Additional corticosteroids or alternative antibiotics for the treatment of macrolide-resistant *Mycoplasma pneumoniae* pneumonia. Korean J Pediatr. (2017) 60:245–7. 10.3345/kjp.2017.60.8.245 29042865 PMC5638721

[40] Chaudhry R Ghosh A Chandolia A. Pathogenesis of *Mycoplasma pneumoniae*: an update. Indian J Med Microbiol. (2016) 34:7–16. 10.4103/0255-0857.174112 26776112

[41] Rhen T Cidlowski JA. Antiinflammatory action of glucocorticoids–new mechanisms for old drugs. N Engl J Med. (2005) 353:1711–23. 10.1056/NEJMra050541 16236742

[42] von HL. Role of persistent infection in the control and severity of asthma: focus on *Chlamydia pneumoniae*. Eur Respir J. (2002) 19:546–56. 10.1183/09031936.02.00254402 11936537

[43] Lee KY Rhim JW Kang JH. Immunopathogenesis of COVID-19 and early immunomodulators. Clin Exp Pediatr. (2020) 63:239–50. 10.3345/cep.2020.00759 32664709 PMC7374000

[44] Yoon IA Hong KB Lee HJ Yun KW Park JY Choi YH. Radiologic findings as a determinant and no effect of macrolide resistance on clinical course of *Mycoplasma pneumoniae* pneumonia. BMC Infect Dis. (2017) 17:402. 10.1186/s12879-017-2500-z 28592263 PMC5463359

[45] Chen YC Hsu WY Chang TH. Macrolide-resistant *Mycoplasma pneumoniae* infections in pediatric community-acquired pneumonia. Emerg Infect Dis. (2020) 26:1382–91. 10.3201/eid2607.200017 32568052 PMC7323531

