# Supplementary material for: Low-Dose Corticosteroid Treatment in Children With Mycoplasma pneumoniae Pneumonia: A Retrospective Cohort Study
Source: Front Pediatr. 2020 Nov 23;8:566371. doi: 10.3389/fped.2020.566371 (PMC7720903; doi:10.3389/fped.2020.566371)
Supplement: Supplementary file 1 [file Data_Sheet_1.docx]

**Appendix table 1.** Characteristics of children with *Mycoplasma pneumoniae* pneumonia stratified by adjunctive corticosteroid treatment after propensity score-matching.

| **Variables** | **Total** | **No-corticosteroid** | **Corticosteroid** | ***P*-value** |
| --- | --- | --- | --- | --- |
|  | **(n = 381)** | **(n = 127)** | **(n = 254)** |  |
| Male, n (%) | 173 (45.4) | 50 (39.4) | 123 (48.4) | 0.094 |
| Age, mean (SD), years | 5.1 (2.7) | 4.9 (2.9) | 5.1 (2.6) | 0.343 |
| Weight, mean (SD), kg | 21.0 (9.5) | 20.7 (10.5) | 21.1 (9.0) | 0.766 |
| Fever duration before admission, median (IQR), days | 7.0 (5.0- 9.0) | 7.0 (5.0-9.0) | 7.0 (5.0-8.0) | 0.819 |
| Hypoxemia, n (%) | 5 (1.3) | 1 (0.8) | 4 (1.6) | 0.524 |
| Neurological symptoms, n (%) | 5 (1.3) | 1 (0.8) | 4 (1.6) | 0.524 |
| Encephalitis, n (%) | 1 (0.3) | 0 (0.0) | 1 (0.4) | 0.479 |
| Rash, n (%) | 2 (0.5) | 0 (0.0) | 2 (0.8) | 0.316 |
| Multilobar infiltrates, n (%) | 305 (81.8) | 100 (82.0) | 205 (81.7) | 0.945 |
| Multi lobar consolidations, n (%) | 100 (26.5) | 31 (24.8) | 69 (27.3) | 0.608 |
| Atelectasis, n (%) | 30 (8.3) | 6 (5.2) | 24 (9.7) | 0.143 |
| Pleural effusion, n (%) | 76 (20.6) | 21 (17.5) | 55 (22.1) | 0.307 |
| SOFA≥1, n (%) | 11 (2.9) | 5 (3.9) | 6 (2.4) | 0.387 |
| Severe pneumonia, n (%) | 77 (20.2) | 21 (16.5) | 56 (22.0) | 0.207 |
| Refractory pneumonia, n(%) | 113 (29.7) | 37 (29.1) | 76 (29.9) | 0.874 |
| White blood cell, median (IQR), 10^9^/L | 7.0 (5.5- 9.5) | 7.0 (5.6-10.4) | 7.0 (5.5- 9.2) | 0.145 |
| Platelet, mean (SD), 10^9^/L | 307.3 (112.7) | 321.6 (117.5) | 300.1 (109.8) | 0.081 |
| CRP, median (IQR), mg/L | 11.0 ( 4.0- 25.0) | 10.0 (4.0-22.0) | 11.0 (4.0-26.0) | 0.368 |
| Lactic dehydrogenase, median (IQR), U/L | 374.0 (342.0- 418.0) | 361.0 (332.5-402.0) | 376.0 (346.0-422.0) | 0.488 |
| Total bilirubin, mean (SD), μmol/L | 5.5 (2.7) | 5.7 (3.4) | 5.4 (2.2) | 0.332 |
| Creatine, mean (SD), μmol/L | 30.3 ( 8.0) | 30.6 (8.3) | 30.1 (7.8) | 0.542 |
| Creatine Kinase Isoenzyme, mean (SD) , U/L | 22.9 (10.3) | 23.4 (10.6) | 22.6 (10.1) | 0.508 |
| Interleukin-10, median (IQR), pg/mL | 5.2 (2.5- 9.6) | 5.0 (2.5- 8.7) | 5.3 (2.5-10.1) | 0.107 |
| **Prognosis** |  |  |  |  |
| Fever duration after admission, median (IQR), days | 2.0 ( 0.0- 3.0) | 1.0 (0.0-2.0) | 2.0 (1.0-3.0) | <0.001 |
| Total fever duration, median (IQR), days | 9.0 (7.0-11.0) | 8.0 (6.0-10.0) | 9.0 (7.0-11.0) | 0.103 |
| Length of hospital stay, median (IQR), days | 7.0 (6.0-9.0) | 6.0 (5.0-8.0) | 8.0 (6.0-9.0) | <0.001 |
| CRP recovery time, median (IQR), days | 4.0 ( 3.0- 6.0) | 4.0 (3.0-5.0) | 5.0 (3.0-6.0) | 0.054 |
| Imaging recovery time, median (IQR), days | 15.0 ( 9.0- 19.0) | 16.0 (13.0-20.5) | 13.5 ( 8.2-18.8) | 0.104 |

*Data are presented as mean(SD) or median(IQR) or n (%).*

*SD, standard deviation; IQR, interquartile range; SOFA, sequential organ failure assessment score; CRP, C-reactive protein.*

**Appendix table 2.** Logistic regression analysis for the effects of adjunctive corticosteroid treatment in *Mycoplasma pneumoniae* pneumonia patients after propensity score-matching.

| **Variables** | **Total** | **No-corticosteroid** | **Corticosteroid** | **OR^a^ (95%CI)** | ***P*-value** |
| --- | --- | --- | --- | --- | --- |
| **Overall** | **n = 381** | **n = 127** | **n = 254** |  |  |
| Fever duration after admission > 75^th^ (3 days) | 71 (25.7) | 14 (18.2) | 57 (28.6) | 1.7 (0.9, 3.4) | 0.104 |
| Total fever duration > 75^th^ (11 days) | 79 (20.7) | 23 (18.1) | 56 (22.0) | 1.2 (0.7, 2.1) | 0.483 |
| Length of hospital stay > 75^th^ (9 days) | 72 (18.9) | 15 (11.8) | 57 (22.4) | 2.0 (1.1, 3.8) | 0.026 |
| CRP recovery time > 75^th^ (6 days) | 35 (20.8) | 7 (15.9) | 28 (22.6) | 1.4 (0.5, 3.6) | 0.498 |
| Imaging recovery time > 75^th^ (19 days) | 41 (26.5) | 14 (36.8) | 27 (23.1) | 0.5 (0.2, 1.2) | 0.136 |
| **Patients with severe pneumonia** | **n = 77** | **n = 21** | **n = 56** |  |  |
| Fever duration after admission > 75^th^ (4 days) | 12 (20.3) | 1 (7.1) | 11 (24.4) | 3.3 (0.3, 31.3) | 0.305 |
| Total fever duration > 75^th^ (12 days) | 17 (22.1) | 3 (14.3) | 14 (25.0) | 1.6 (0.4, 6.9) | 0.531 |
| Length of hospital stay > 75^th^ (10 days) | 19 (24.7) | 3 (14.3) | 16 (28.6) | 2.3 (0.6, 9.3) | 0.228 |
| CRP recovery time > 75^th^ (6 days) | 12 (26.7) | 3 (25.0) | 9 (27.3) | 1.0 (0.2, 4.7) | 0.975 |
| Imaging recovery time > 75^th^ (18 days) | 10 (25.0) | 2 (33.3) | 8 (23.5) | 0.6 (0.1, 4.4) | 0.649 |
| **Patients with refractory pneumonia** | **n = 113** | **n = 37** | **n = 76** |  |  |
| Fever duration after admission > 75^th^ (5 days) | 19 (19.6) | 2 (6.7) | 17 (25.4) | 5.0 (1.0, 24.3) | 0.045 |
| Total fever duration > 75^th^ (14 days) | 25 (22.1) | 4 (10.8) | 21 (27.6) | 3.3 (1.0, 10.7) | 0.049 |
| Length of hospital stay > 75^th^ (11 days) | 23 (20.4) | 3 (8.1) | 20 (26.3) | 3.7 (1.0, 14.4) | 0.056 |
| CRP recovery time > 75^th^ (6 days) | 17 (26.2) | 4 (21.1) | 13 (28.3) | 1.4 (0.4, 5.9) | 0.610 |
| Imaging recovery time > 75^th^ (21 days) | 9 (20.5) | 1 (11.1) | 8 (22.9) | 7.1 (0.6, 83.1) | 0.119 |
| **Patients with CRP > 75^th^ (25 mg/L)** | **n = 95** | **n = 30** | **n = 65** |  |  |
| Fever duration after admission > 75^th^ (4 days) | 12 (14.0) | 3 (12.0) | 9 (14.8) | 1.3 (0.3, 5.6) | 0.687 |
| Total fever duration > 75^th^ (11 days) | 21 (22.1) | 4 (13.3) | 17 (26.2) | 2.7 (0.8, 9.4) | 0.116 |
| Length of hospital stay > 75^th^ (10 days) | 19 (20.0) | 5 (16.7) | 14 (21.5) | 1.8 (0.5, 6.4) | 0.357 |
| CRP recovery time > 75^th^ (6 days) | 19 (24.7) | 3 (13.6) | 16 (29.1) | 2.7 (0.7, 10.8) | 0.163 |
| Imaging recovery time > 75^th^ (20 days) | 9 (22.0) | 0 (0.0) | 9 (26.5) | inf. (0.0, Inf) | 0.994 |
| **Patients with LDH > 75^th^ (418 U/L)** | **n = 94** | **n = 26** | **n = 68** |  |  |
| Fever duration after admission > 75^th^ (3 days) | 23 (29.5) | 2 (10.5) | 21 (35.6) | 4.5 (0.9, 21.9) | 0.064 |
| Total fever duration > 75^th^ (13 days) | 20 (21.3) | 5 (19.2) | 15 (22.1) | 1.0 (0.3, 3.4) | 0.972 |
| Length of hospital stay > 75^th^ (9 days) | 19 (20.2) | 4 (15.4) | 15 (22.1) | 1.3 (0.4, 4.6) | 0.683 |
| CRP recovery time > 75^th^ (6 days) | 8 (15.7) | 1 (11.1) | 7 (16.7) | 1.4 (0.1, 14.8) | 0.790 |
| Imaging recovery time > 75^th^ (20 days) | 10 (27.0) | 5 (62.5) | 5 (17.2) | 0.1 (0.0, 0.7) | 0.018 |
| **Patients with IL-10 > 75^th^ (9.6 pg/mL)** | **n = 60** | **n = 15** | **n = 45** |  |  |
| Fever duration after admission > 75^th^ (3 days) | 13 (25.5) | 1 (7.7) | 12 (31.6) | 5.9 (0.7, 53.2) | 0.112 |
| Total fever duration > 75^th^ (13 days) | 14 (23.3) | 4 (26.7) | 10 (22.2) | 0.7 (0.2, 2.9) | 0.625 |
| Length of hospital stay > 75^th^ (9 days) | 9 (15.0) | 1 (6.7) | 8 (17.8) | 2.1 (0.2, 20.0) | 0.526 |
| CRP recovery time > 75^th^ (6 days) | 7 (25.9) | 1 (14.3) | 6 (30.0) | 2.2 (0.2, 25.7) | 0.519 |
| Imaging recovery time > 75^th^ (18 days) | 6 (26.1) | 3 (42.9) | 3 (18.8) | 0.8 (0.1, 6.6) | 0.804 |
| **Patients with pleural effusion** | **n = 76** | **n = 21** | **n = 55** |  |  |
| Fever duration after admission > 75^th^ (4 days) | 11 (18.3) | 1 (6.7) | 10 (22.2) | 3.5 (0.4, 30.7) | 0.259 |
| Total fever duration > 75^th^ (12 days) | 15 (19.7) | 3 (14.3) | 12 (21.8) | 1.5 (0.4, 6.4) | 0.581 |
| Length of hospital stay > 75^th^ (11 days) | 16 (21.1) | 3 (14.3) | 13 (23.6) | 1.7 (0.4, 7.0) | 0.439 |
| CRP recovery time > 75^th^ (6 days) | 12 (27.9) | 3 (25.0) | 9 (29.0) | 1.0 (0.2, 4.7) | 0.952 |
| Imaging recovery time > 75^th^ (18 days) | 11 (26.8) | 1 (16.7) | 10 (28.6) | 2.1 (0.2, 21.4) | 0.520 |
| **Patients with multilobar consolidations** | **n = 100** | **n = 31** | **n = 69** |  |  |
| Fever duration after admission > 75^th^ (3 days) | 24 (31.2) | 5 (23.8) | 19 (33.9) | 1.5 (0.4, 4.9) | 0.531 |
| Total fever duration > 75^th^ (12 days) | 22 (22.0) | 4 (12.9) | 18 (26.1) | 2.2 (0.6, 7.4) | 0.224 |
| Length of hospital stay > 75^th^ (9 days) | 19 (19.0) | 4 (12.9) | 15 (21.7) | 2.2 (0.6, 7.9) | 0.236 |
| CRP recovery time > 75^th^ (6 days) | 10 (18.5) | 5 (41.7) | 5 (11.9) | 0.2 (0.0, 1.0) | 0.051 |
| Imaging recovery time > 75^th^ (20 days) | 11 (26.2) | 3 (23.1) | 8 (27.6) | 1.6 (0.3, 8.1) | 0.574 |

*Data are presented as no. (%), OR and 95% CI.*

*OR, odds ratio; CI, confidence interval; CRP, C-reactive protein; LDH, lactic* *dehydrogenase; IL-10, Interleukin-10.*

*^a^ Adjusted for age, sex, and severe pneumonia.*

**Appendix table 3.** Logistic regression analysis for the effects of adjunctive corticosteroid treatment stratified by medication time in *Mycoplasma pneumoniae* pneumonia patients after propensity score-matching.

|  | **No-corticosteroid** | **Corticosteroid (1 – 5 days)** | | | **Corticosteroid (6 – 10 days)** | | | **Corticosteroid ( > 10 days)** | | |
| --- | --- | --- | --- | --- | --- | --- | --- | --- | --- | --- |
| **Variables** | **（n = 127）** | **（n = 41）** | | | **（n = 155）** | | | **（n = 47）** | | |
|  | **n (%)** | **n (%)** | **OR^a^ (95%CI)** | *P*-**value** | **n (%)** | **OR (95%CI)** | *P*-**value** | **n (%)** | **OR (95%CI)** | *P*-**value** |
| Fever duration after admission > 75^th^ (3 days) | 14 (18.2) | 9 (29.0) | 1.9 (0.7, 5.0) | 0.218 | 33 (25.6) | 1.5 (0.8, 3.1) | 0.234 | 13 (37.1) | 2.5 (1.0, 6.2) | 0.058 |
| Total fever duration > 75^th^ (11 days) | 23 (18.1) | 7 (17.1) | 0.9 (0.3, 2.2) | 0.784 | 18 (11.6) | 0.6 (0.3, 1.2) | 0.121 | 28 (59.6) | 6.5 (3.1, 13.6) | <0.001 |
| Length of hospital stay > 75^th^ (9 days) | 15 (11.8) | 5 (12.2) | 0.9 (0.3, 2.8) | 0.909 | 37 (23.9) | 2.3 (1.2, 4.6) | 0.012 | 14 (29.8) | 3.0 (1.3, 6.9) | 0.012 |
| CRP recovery time > 75^th^ (6 days) | 7 (15.9) | 2 (11.1) | 0.7 (0.1, 3.8) | 0.673 | 18 (23.4) | 1.4 (0.5, 3.9) | 0.470 | 8 (32.0) | 2.3 (0.7, 7.7) | 0.193 |
| Imaging recovery time > 75^th^ (19 days) | 14 (36.8) | 3 (33.3) | 0.9 (0.2, 4.1) | 0.857 | 18 (22.5) | 0.5 (0.2, 1.2) | 0.138 | 5 (21.7) | 0.5 (0.2, 1.7) | 0.271 |

*Data are presented as no. (%), OR, and 95% CI.*

*OR, odds ratio; CI, Confidence interval; CRP, C-reactive protein.*

***^a^*** *Adjusted for age, sex, and severe pneumonia.*
